# Quality of Inpatient Pediatric Case Management for Four Leading Causes of Child Mortality at Six Government-Run Ugandan Hospitals

**DOI:** 10.1371/journal.pone.0127192

**Published:** 2015-05-19

**Authors:** David Sears, Arthur Mpimbaza, Ruth Kigozi, Asadu Sserwanga, Michelle A. Chang, Bryan K. Kapella, Steven Yoon, Moses R. Kamya, Grant Dorsey, Theodore Ruel

**Affiliations:** 1 Department of Medicine, San Francisco General Hospital, University of California San Francisco, San Francisco, CA, United States of America; 2 Uganda Malaria Surveillance Project, Kampala, Uganda; 3 Child Health & Development Centre, Makerere University, Kampala, Uganda; 4 Malaria Branch, Centers for Disease Control and Prevention, Atlanta, GA, United States of America; 5 United States President’s Malaria Initiative, Centers for Disease Control and Prevention, Atlanta, GA, United States of America; 6 Department of Medicine, Makerere University College of Health Sciences, Kampala, Uganda; 7 Department of Pediatrics, University of California San Francisco, San Francisco, CA, United States of America; University College London, UNITED KINGDOM

## Abstract

**Background:**

A better understanding of case management practices is required to improve inpatient pediatric care in resource-limited settings. Here we utilize data from a unique health facility-based surveillance system at six Ugandan hospitals to evaluate the quality of pediatric case management and the factors associated with appropriate care.

**Methods:**

All children up to the age of 14 years admitted to six district or regional hospitals over 15 months were included in the study. Four case management categories were defined for analysis: suspected malaria, selected illnesses requiring antibiotics, suspected anemia, and diarrhea. The quality of case management for each category was determined by comparing recorded treatments with evidence-based best practices as defined in national guidelines. Associations between variables of interest and the receipt of appropriate case management were estimated using multivariable logistic regression.

**Results:**

A total of 30,351 admissions were screened for inclusion in the analysis. Ninety-two percent of children met criteria for suspected malaria and 81% received appropriate case management. Thirty-two percent of children had selected illnesses requiring antibiotics and 89% received appropriate antibiotics. Thirty percent of children met criteria for suspected anemia and 38% received appropriate case management. Twelve percent of children had diarrhea and 18% received appropriate case management. Multivariable logistic regression revealed large differences in the quality of care between health facilities. There was also a strong association between a positive malaria diagnostic test result and the odds of receiving appropriate case management for comorbid non-malarial illnesses - children with a positive malaria test were more likely to receive appropriate care for anemia and less likely for illnesses requiring antibiotics and diarrhea.

**Conclusions:**

Appropriate management of suspected anemia and diarrhea occurred infrequently. Pediatric quality improvement initiatives should target deficiencies in care unique to each health facility, and interventions should focus on the simultaneous management of multiple diagnoses.

## Introduction

The leading causes of childhood death in sub-Saharan Africa—malaria, pneumonia, malnutrition, and diarrhea—are both treatable and preventable, yet one in ten children do not survive until their fifth birthday [1,2]. While the causes of mortality are multifactorial and complex, poor quality inpatient medical care likely contributes to a significant proportion of child deaths [3–12]. In Uganda, 75% of children who die receive treatment in a health facility for the illness that led to death and approximately 40% of child deaths occur in health facilities [13]. While studies have suggested that care of children in Ugandan health facilities often falls short of internationally accepted best practices [3,14–16], a more complete understanding of the quality of case management of hospitalized Ugandan children and the factors that predict inappropriate practices is needed to guide quality improvement efforts.

In 2010, the Uganda Malaria Surveillance Project (UMSP) and the National Malaria Control Program (NMCP) created a health facility-based surveillance program to prospectively track trends in disease burden, treatment practices, and clinical outcomes of pediatric inpatients at six government-run Ugandan hospitals. UMSP comprises four main components: 1) implementation of a standardized medical record form (MRF) to prospectively capture data on all pediatric admissions, 2) training in malaria case management conducted at the time of MRF implementation, 3) emphasis on malaria diagnosis by laboratory confirmation, and 4) periodic review of collected malaria data with each health facility to facilitate discussions on how to improve data quality and treatment practices. While the program initially focused on malaria surveillance, high-quality data on non-malarial illness was also collected.

Our aim with this analysis was to utilize data from UMSP to evaluate the quality of inpatient pediatric care at participating hospitals across a range of illnesses. As a demonstration project we focused our analysis on four case management categories: diagnosis and management of suspected malaria, antibiotic usage for selected illnesses (pneumonia, malnutrition, sepsis, meningitis, and tetanus), diagnosis and management of suspected anemia, and management of diarrhea. Quality care was defined with reference to evidence-based best practices promoted by the Uganda Ministry of Health and published in *Uganda Clinical Guidelines* [17], as well as the World Health Organization (WHO) *Pocket Book of Hospital Care for Children* [18]. After reporting on the proportion of children receiving quality care for selected conditions, we then sought to determine whether there was heterogeneity in quality between health facilities, how quality changed over time, and if certain diseases and patient characteristics were associated with a higher probability of receiving better care. The ultimate goal of this analysis was to identify targets for quality improvement interventions in Uganda and similar settings.

## Methods

### Health facilities and patient care

**Fig 1:**
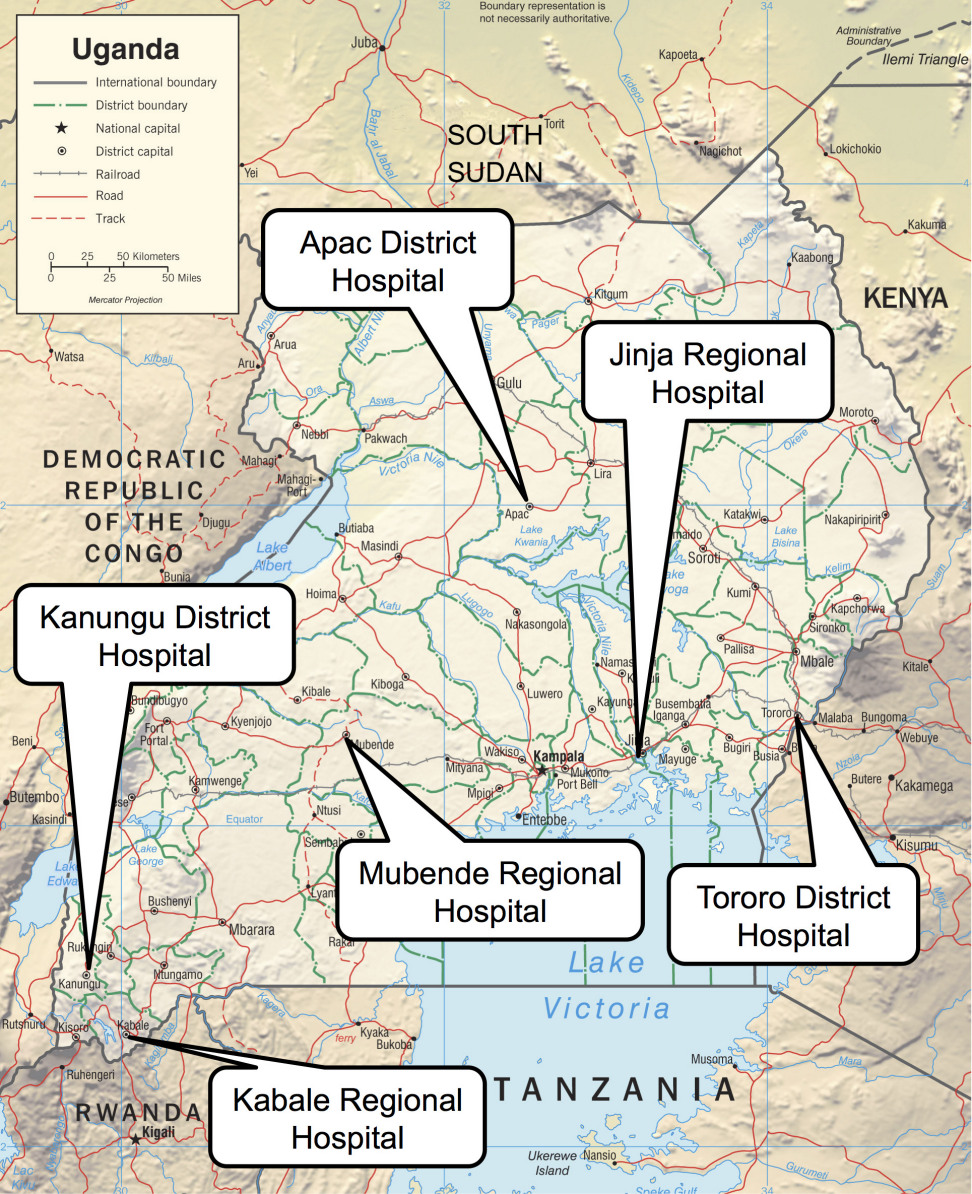
Location of the six hospitals participating in the UMSP health facility-based surveillance program [Image adapted from the original (source: https://www.cia.gov/library/publications/cia-maps-publications/map-downloads/uganda-physiog.jpg/image.jpg)].

The UMSP health facility-based inpatient surveillance program was implemented between 2010 and 2011 at six health facilities in Uganda (Fig 1). Among the six health facilities, three were district hospitals located in Tororo, Kanungu, and Apac, and three were regional referral hospitals located in Jinja, Kabale, and Mubende. The hospitals were selected to represent regions of high (Tororo and Apac), medium (Jinja and Mubende), and low (Kanungu and Kabale) malaria transmission intensity. At all hospitals except Jinja, clinical officers (diploma level training in medicine) and nurses made the decision to admit patients and develop the initial plan of care. On the wards, nurses reviewed admitted children and clinical officers or medical doctors periodically reviewed critically ill children. At Jinja, intern doctors were responsible for assessing and reviewing children on a daily basis and pediatricians supervised their care. Clinical officers were also involved in patient care at Jinja, mainly through admissions from the outpatient department. Facilities at each hospital included surgical suites, a pharmacy, and a laboratory. Diagnostic capabilities, which were not always available, included malaria blood smears, hemoglobin measurement, HIV antibody testing, and blood typing. Radiographs were not commonly available at any participating health facility. Essential medications and supplies were distributed to health facilities through the government-run National Medical Stores, although stock-outs did occur. Supplemental treatments in the form of oxygen, blood transfusions, and nutritional rehabilitation were available sporadically.

### Data management

UMSP surveillance data were collected on an individual-level, using the standardized medical record form (MRF) (S1 Appendix), which replaced hospital charts at each health facility. The MRF was completed by clinicians and captured basic demographic information, presenting signs and symptoms, diagnostic testing, diagnoses rendered upon admission and discharge, treatments prescribed and administered, and outcomes at the end of hospitalization (death, discharge, referral, or left prior to discharge). Where applicable, check boxes were used to minimize transcription errors, promote completeness, and facilitate data analysis. Following discharge, data from the MRF of each patient were entered into an Access database (Microsoft Corporation, Redmond, WA) by a data officer at each health facility. At the end of each month all data were sent to a secure server in Kampala. Data were checked for errors and then merged into a relational database.

### Program implementation and health facility support

Prior to the implementation of the surveillance program at each of the six health facilities, hospital staff received a two-day training on the use of the new MRF and the importance of clear documentation and data quality in relation to disease surveillance. Training also focused on the basics of malaria case management with an emphasis on the use of diagnostic testing, withholding antimalarial medications for patients with a negative diagnostic test, and investigating non-malarial causes of febrile illness after a negative diagnostic test. Following the implementation of surveillance, each health facility received one-day follow-up visits two to three times a year. At these visits data were shared with clinicians and hospital administrators regarding malaria case management, all-cause inpatient mortality, and data quality. Malaria case management focused on increasing the utilization of diagnostic testing. Staff at each health facility were able to see how their data compared to those from the other five participating health facilities and discussions were held about how to improve data quality and treatment practices. In addition to these workshops, each health facility also received laboratory support for malaria blood smears in the form of a steady supply of reagents and slides, additional training for microscopists, and microscopy quality assurance.

### Statistical analysis and case management definitions

All children under the age of 14 years admitted from October 1, 2012 to December 31, 2013 were screened for inclusion in the analysis. October of 2012 was selected as the start of the study as this date included data captured following modifications to the MRF and allowed the health facilities to have at least one year of experience with the new surveillance system. Four case management categories were selected for analysis: suspected malaria, illnesses requiring antibiotics (pneumonia, malnutrition, sepsis, meningitis, and tetanus), suspected anemia, and diarrhea. These categories were selected as they 1) represent common high burden diseases and clinical presentations encountered in the hospitals, 2) were associated with evidence-based case management practices that were recommended in *Uganda Clinical Guidelines* or the WHO *Pocket Book of Hospital Care for Children*, and 3) were easily captured by the surveillance system. A summary of the similarities and differences between the based best practices used in this study (which are defined subsequently) and the best practices defined by WHO and Uganda clinical guidelines is outlined in Table 1.

**Table 1:** Summary of case management best practices defined by Uganda/WHO clinical guidelines and best practices used in this analysis.

Case management category	Subcategory	Uganda/WHO best practice^a^	Best practice used in this analysis
**Suspected malaria**	Fever	1) If available, blood smear or RDT for malaria, urinalysis, and blood culture; 2) comprehensive history and physical to determine cause of fever; 3) paracetamol for temperature ≥ 39°C if age ≥ 2 months	Blood smear or RDT for malaria
	Negative malaria test	1) No antimalarial	No antimalarial
	Severe malaria	1) Properly classify as severe malaria; 2) parenteral artesunate (preferred), artemether, or quinine	Parenteral artesunate, artemether, or quinine
	Uncomplicated malaria	1) Properly classify as uncomplicated malaria; 2) artemisinin-based combination therapy (ACT)	ACT or parenteral artesunate, artemether, or quinine
**Selected illnesses requiring antibiotics**	Pneumonia	1) Classify as severe or uncomplicated; 2) provide oxygen if oxygen saturation <90%; 3) chest x-ray if available; 4) antibiotics (regimen dependent on severity and age of child)	Any antibiotic
	Malnutrition	1) Comprehensive history and physical to determine cause of malnutrition; 2) antibiotics (regimen dependent on severity); 3) assess for vitamin A deficiency, HIV infection, and parasitic worms; treat as indicated; 4) correct micronutrient deficiencies; 5) initiate appropriate feeding regimen	Any antibiotic
	Sepsis	1) Comprehensive history and physical to determine cause of sepsis; 2) if available, blood smear or RDT for malaria, urinalysis, blood culture, and chest x-ray; 3) antibiotics (regimen dependent on suspected source and age of child)	Any antibiotic
	Meningitis	1) Perform lumbar puncture unless signs of elevated intracranial pressure; 2) antibiotics (regimen dependent on lumbar puncture and age of child)	Ceftriaxone, chloramphenicol, gentamicin, ampicillin, or penicillin
	Tetanus	1) Clean wounds, remove necrotic tissue; 2) penicillin or metronidazole; tetanus immunoglobulin or antitoxin	Penicillin, metronidazole, or chloramphenicol
**Suspected anemia**	Pallor	1) Hemoglobin testing	Hemoglobin testing
	Jaundice	1) If available, complete blood count and bilirubin; 2) consider liver ultrasound and additional hemolysis labs as indicated	Hemoglobin testing
	Sickle cell disease	1) Hemoglobin testing	Hemoglobin testing
	Severe anemia	1) Comprehensive history and physical to determine cause; 2) blood transfusion	Blood transfusion
**Diarrhea**	Diarrhea	1) Classify severity of dehydration; 2) ORS^b^ or intravenous fluids (dependent on severity, age of child, malnutrition); 3) zinc supplementation	ORS or intravenous fluids and zinc supplementation
	Dysentery	1) Nalidixic acid, cotrimoxazole, or ceftriaxone	Nalidixic acid, cotrimoxazole, or ceftriaxone

^a^ Uganda/WHO best practices also frequently call for the assessment and treatment of comorbidities such as hypoglycemia, anemia, dehydration, and respiratory distress

^b^ Oral rehydration therapy

Patients were included in the suspected malaria case management category if they reported a history of fever or had a measured temperature ≥ 38.0°C. Individual evidence-based best practices included the following: 1) obtaining a malaria blood smear or rapid diagnostic test (RDT), 2) withholding antimalarials if the blood smear or RDT was negative, 3) treating severe malaria with an appropriate antimalarial, and 4) treating uncomplicated malaria with an appropriate antimalarial. Malaria was defined as having a positive blood smear or RDT. Severe malaria was defined as having malaria plus any documentation on the MRF that indicated the child met WHO clinical or laboratory criteria for severe disease [19]. Appropriate antimalarial medication for severe malaria included receipt of any parenteral artesunate, artemether, or quinine. While Ugandan and WHO guidelines define appropriate antimalarial medication for uncomplicated malaria as the receipt of artemisinin-based combination therapy (ACT), we also defined any parenteral artesunate, artemether, or quinine as appropriate therapy as these medications are also highly efficacious. Dosing information was not captured by the MRF and was thus not considered in the assessment of appropriate care. A binary composite indicator of quality case management was then generated for each suspected malaria case, requiring fulfillment of all of the following: a blood smear or RDT followed by either 1) withholding antimalarial treatment after a negative test result, 2) treating laboratory-confirmed severe malaria with any parenteral artesunate, artemether, or quinine, or 3) treating laboratory-confirmed uncomplicated malaria with any ACT or parenteral artesunate, artemether, or quinine.

Patients were included in the selected illnesses requiring antibiotics category if their final discharge diagnoses included pneumonia, sepsis, meningitis, or tetanus, or if they had severe malnutrition defined as weight for age z-score of < -3 (an objective classification of malnutrition was utilized due to the under diagnosis of this condition). To determine appropriate management, discharge diagnoses were felt to be more reliable than admission diagnoses as the latter were usually rendered by the clinician in the outpatient clinic based only on information from the initial stages of evaluation. Admission diagnoses were often changed soon after hospitalization once additional information, such as malaria diagnostic testing, became available. Given the difficulty in determining appropriate antibiotics for each of these conditions from the surveillance system alone, individual evidence-based use of antibiotics was defined broadly. Any antibiotics were considered appropriate for pneumonia, malnutrition, and sepsis. Since cerebrospinal fluid analysis, if performed, was not captured by the MRF, appropriate antibiotics for meningitis included any parenteral antibiotic with meningeal penetration (ceftriaxone, chloramphenicol, gentamicin, ampicillin, and penicillin). Appropriate antibiotics for tetanus included parenteral penicillin, metronidazole, or chloramphenicol. As with antimalarials, dosing information was not captured and could not be considered in the assessment of appropriate care. A binary composite indicator of quality care was also generated to assess the overall quality of antibiotic selection. Patients were classified as having received appropriate management if they received appropriate antibiotics for all of the selected illnesses with which they were diagnosed.

The suspected anemia case management category comprised every child with pallor or jaundice on physical examination or a diagnosis of sickle cell disease. Individual evidence-based best practices included hemoglobin testing and blood transfusion for severe anemia. Severe anemia was defined as hemoglobin level < 5.0g/dl. To be classified as appropriate, the binary composite indicator of quality care required both hemoglobin testing and transfusion if testing revealed severe anemia.

The diarrhea case management category involved every child with a clinical diagnosis of diarrhea or dysentery. Individual evidence-based best practices included receipt of oral rehydration solution (ORS) or intravenous fluids, receipt of zinc supplementation, and appropriate antibiotics for patients with dysentery. Antibiotics for dysentery were defined as receipt of nalidixic acid, cotrimoxazole, or ceftriaxone, based on what is provided to the health facilities. The composite indicator of quality care for diarrhea required that each patient receive ORS or intravenous fluids as well as zinc and that each patient with dysentery additionally receive appropriate antibiotics.

The proportion of eligible patients receiving appropriate management for each evidence-based best practice and the composite indicators of quality care for each case management category were calculated. Potential factors associated with receiving appropriate care for each composite indicator were evaluated using univariable and multivariable logistic regression models. Exposure variables of interest included health facility, gender, age, duration of hospitalization, weekday or weekend admission, and presence of comorbid malaria or bacterial infection (defined, as above, as a discharge diagnosis of pneumonia, sepsis, meningitis, tetanus, or severe malnutrition). To evaluate the changes in case management composite indicators over calendar time, univariable logistic regression analysis was also performed using three-month time intervals as the exposure variable of interest (stratified by health facility). Because malaria transmission and the absolute number of inpatient admissions were stable throughout the year at each site, intervals were based on calendar time as opposed to seasonality. Statistical analysis was performed using Stata 12.0 (Stata Corp, College Station, TX). A p-value of <0.05 was considered statistically significant.

### Ethics statement

The UMSP sentinel site surveillance system collects routine health information to supplement Ugandan’s Health Management Information System and all patient data are anonymized and de-identified prior to entry. The authors do not have access to identifying information prior to data entry and do not interact with patients. Consequently, the surveillance system has been deemed nonresearch by the Center for Global Health at the Centers for Disease Control and Prevention (CDC) (tracking number 2014–205). This determination was made because the primary intent of data collection is public health practice or disease control, specifically routine surveillance activities. The determination is congruent with the CDC Policy “Distinguishing Public Health Research and Public Health Nonresearch” [20]. Institutional Review Board approval and informed consent were not deemed necessary for this analysis.

## Results

There were a total of 30,351 admissions of children under the age of 14 during the fifteen-month study period (range 1,557–9,103 across the health facilities). Fewer than half of all admissions were female (46%). The median age of admitted children was 20 months, with 84% under 5 years old, and 28% under 1 year old. The median duration of hospital stay was three days (inter-quartile range 2–4 days). The inpatient mortality rate was 3%, ranging from 1% in Kanungu and Apac to 5% in Jinja (Table 2). Incomplete records were rare, with at least one symptom, physical exam finding, diagnosis, and treatment captured by the MRF in 99% of admissions. Among all admissions, 29,338 (97%) met criteria for inclusion into at least one case management category.

**Table 2:** Characteristics of pediatric admissions, overall and by health facility.

Variable	All Sites	Jinja	Kanungu	Apac	Mubende	Tororo	Kabale
Admissions, n (% total)	30,351	9,103 (30%)	1,557 (5%)	4,304 (14%)	6,045 (20%)	7,457 (25%)	1,885 (6%)
Female gender, n (%)	13,907 (46%)	4,089 (45%)	717 (46%)	2,010 (47%)	2,723 (45%)	3,465 (46%)	903 (48%)
Age in months, median (IQR^a^)	20 (10–36)	18 (9–36)	24 (12–59)	29 (16–48)	20 (11–36)	18 (9–34)	15 (8–27)
Length of stay in days, median (IQR)	3 (2–4)	3 (2–4)	2 (2–3)	3 (2–4)	4 (2–5)	3 (2–4)	3 (2–6)
Positive malaria diagnostic test, n (%)	14,249 (47%)	4,195 (46%)	587 (38%)	2,677 (62%)	2,670 (44%)	4,022 (54%)	98 (5%)
Deaths, n (%)	802 (3%)	415 (5%)	15 (1%)	53 (1%)	123 (2%)	120 (2%)	76 (4%)
Suspected malaria, n (%)	27,809 (92%)	8,552 (94%)	1,412 (91%)	4,004 (93%)	5,449 (90%)	6,920 (93%)	1,472 (78%)
Selected illnesses requiring antibiotics, n (%)	9,584 (32%)	3,027 (33%)	335 (22%)	869 (20%)	2,308 (38%)	1,857 (25%)	1,188 (63%)
Suspected anemia, n (%)	9,162 (30%)	4,199 (46%)	95 (6%)	279 (6%)	2,375 (39%)	1,803 (24%)	411 (22%)
Diarrhea, n (%)	3,606 (12%)	664 (7%)	192 (12%)	194 (5%)	678 (11%)	1524 (20%)	354 (19%)

^a^ Inter-quartile range

**Fig 2:**
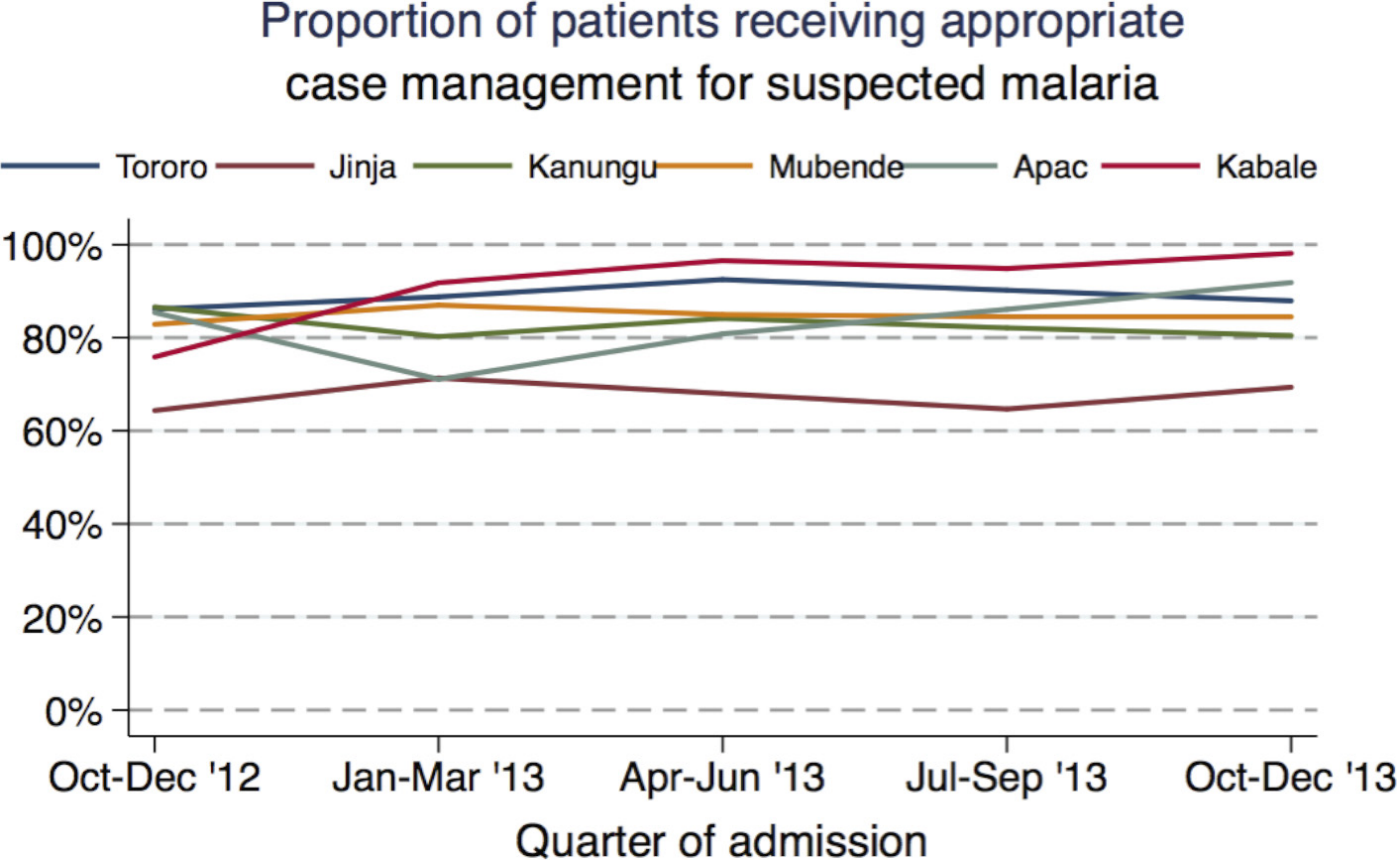
Trends in appropriate inpatient case management of suspected malaria, by health facility.

Suspected malaria was present in most patients (92%; range 78–94% across the health facilities). Nearly all patients (97%) who had suspected malaria received a laboratory test for malaria (Table 3). Among the 27,083 children tested for malaria, 13,143 (49%) tested negative. Antimalarial medications were withheld in 71% of patients who had a negative diagnostic test. Among those receiving an antimalarial after a negative diagnostic test, 84% received more than one dose. Among the 13,940 children who tested positive for malaria, 10,116 (73%) had severe malaria. An appropriate antimalarial was given to 93% of all patients with severe malaria and 97% of all patients with uncomplicated malaria. Eighty one percent of patients with suspected malaria were managed appropriately per the composite indicator of quality care. Receipt of antimalarial medications after a negative malaria test accounted for 71% of the failure to manage patients appropriately in this category. The proportion of children with appropriate management of suspected malaria varied considerably by health facility, with rates ranging from 68% in Jinja to 93% in Kabale (Table 4). Children < 1 year of age and ages 1 to < 5 years were slightly more likely to receive appropriate management for suspected malaria than children ≥ 5 years (82% and 81% vs. 78% respectively; p<0.01 for each comparison). Children who were hospitalized for < 1 day were less likely to receive appropriate management for suspected malaria compared to those with a longer duration of hospitalization (67% vs. 82%; p<0.01). Those admitted on a weekend were slightly less likely to receive appropriate care (78% vs. 82%; p<0.01). Changes in the quality of suspected malaria case management at three-month intervals for each health facility are presented in Fig 2. Significant changes measured over time were only noted in two health facilities, both of which experienced improvement: Apac (OR 1.23 per three-month interval; 95% CI 1.15–1.30; p <0.01) and Kabale (OR 2.10; 95% CI 1.74–2.53; p<0.01).

**Table 3:** Inpatient case management best practices.

Case management category	Evidence-based best practice	Received (%)
**Suspected malaria**	Blood smear or RDT^a^ for malaria if febrile	27,083/27,809 (97%)
	No antimalarial if negative malaria test	9,383/13,143 (71%)
	Appropriate antimalarial if severe malaria	9,432/10,116 (93%)
	Appropriate antimalarial if uncomplicated malaria	3,700/3,824 (97%)
	**Composite indicator of quality care received (%)**	**22,515/27,809 (81%)**
**Selected illnesses requiring antibiotics**	Antibiotics if pneumonia	4,537/4,715 (96%)
	Antibiotics if severe malnutrition	2,550/3,293 (77%)
	Antibiotics if sepsis	2,243/2,342 (96%)
	Antibiotics with meningeal penetration if meningitis	92/111 (83%)
	Appropriate antibiotics if tetanus	37/70 (53%)
	**Composite indicator of quality care received (%)**	**8,550/9,584 (89%)**
**Suspected anemia**	Hemoglobin testing if pallor	4,161/8,799 (47%)
	Hemoglobin testing if jaundice	310/664 (47%)
	Hemoglobin testing if sickle cell disease	291/546 (53%)
	Blood transfusion if hemoglobin < 5g/dl	1,597/2,405 (66%)
	**Composite indicator of quality care received (%)**	**3,461/9,162 (38%)**
**Diarrhea**	ORS^b^ or intravenous fluids if diarrhea	2262/3606 (63%)
	Zinc supplementation if diarrhea	870/3606 (24%)
	Appropriate antibiotics if dysentery	42/87 (48%)
	**Composite indicator of quality care received (%)**	**649/3,606 (18%)**

^a^ Rapid diagnostic test

^b^ Oral rehydration solution

**Table 4:** Associations between characteristics and receipt of appropriate inpatient case management for suspected malaria.

Category	Co-variable	Appropriate management (%)	Univariable		Multivariable	
			OR (95% CI)	p-value	OR (95% CI)	p-value
	Jinja	5,783/8,552 (68%)	reference	—	reference	—
	Kanungu	1,168/1,412 (83%)	2.29 (1.98–2.65)	<0.01	2.34 (2.02–2.71)	<0.01
Health facility	Apac	3,390/4,004 (85%)	2.64 (2.40–2.91)	<0.01	2.59 (2.35–2.86)	<0.01
	Mubende	4,618/5,449 (85%)	2.66 (2.44–2.90)	<0.01	2.65 (2.42–2.89)	<0.01
	Tororo	6,184/6,920 (89%)	4.02 (3.68–4.40)	<0.01	3.89 (3.56–4.25)	<0.01
	Kabale	1,372/1,472 (93%)	6.57 (5.34–8.09)	<0.01	6.51 (5.28–8.03)	<0.01
Gender	Male	12,143/15,020 (81%)	reference	—	reference	—
	Female	103,72/12,789 (81%)	1.02 (0.96–1.08)	0.59	1.01 (0.95–1.07)	0.83
Age	≥ 5 years	3,309/4,227 (78%)	reference	—	reference	—
	1 - < 5 years	13,069/16,064 (81%)	1.21 (1.11–1.32)	<0.01	1.21 (1.11–1.32)	<0.01
	< 1 year	6,137/7,518 (82%)	1.23 (1.12–1.35)	<0.01	1.29 (1.17–1.43)	<0.01
Duration of hospitalization	> 1 day	19,811/24,222 (82%)	reference	—	reference	—
	1 day	1,980/2,512 (79%)	0.83 (0.75–0.92)	<0.01	0.96 (0.86–1.07)	0.44
	< 1 day	724/1,075 (67%)	0.46 (0.40–0.52)	<0.01	0.55 (0.48–0.63)	<0.01
Day of admission	Weekday	18,142/22,191 (82%)	reference	—	reference	—
	Weekend	4,373/5,618 (78%)	0.78 (0.73–0.84)	<0.01	0.82 (0.76–0.88)	<0.01
Comorbid bacterial infection	Absent	15,445/19,078 (81%)	reference	—	reference	—
	Present	7,070/8,731 (81%)	1.00 (0.94–1.07)	0.97	0.97 (0.90–1.04)	0.36

**Fig 3:**
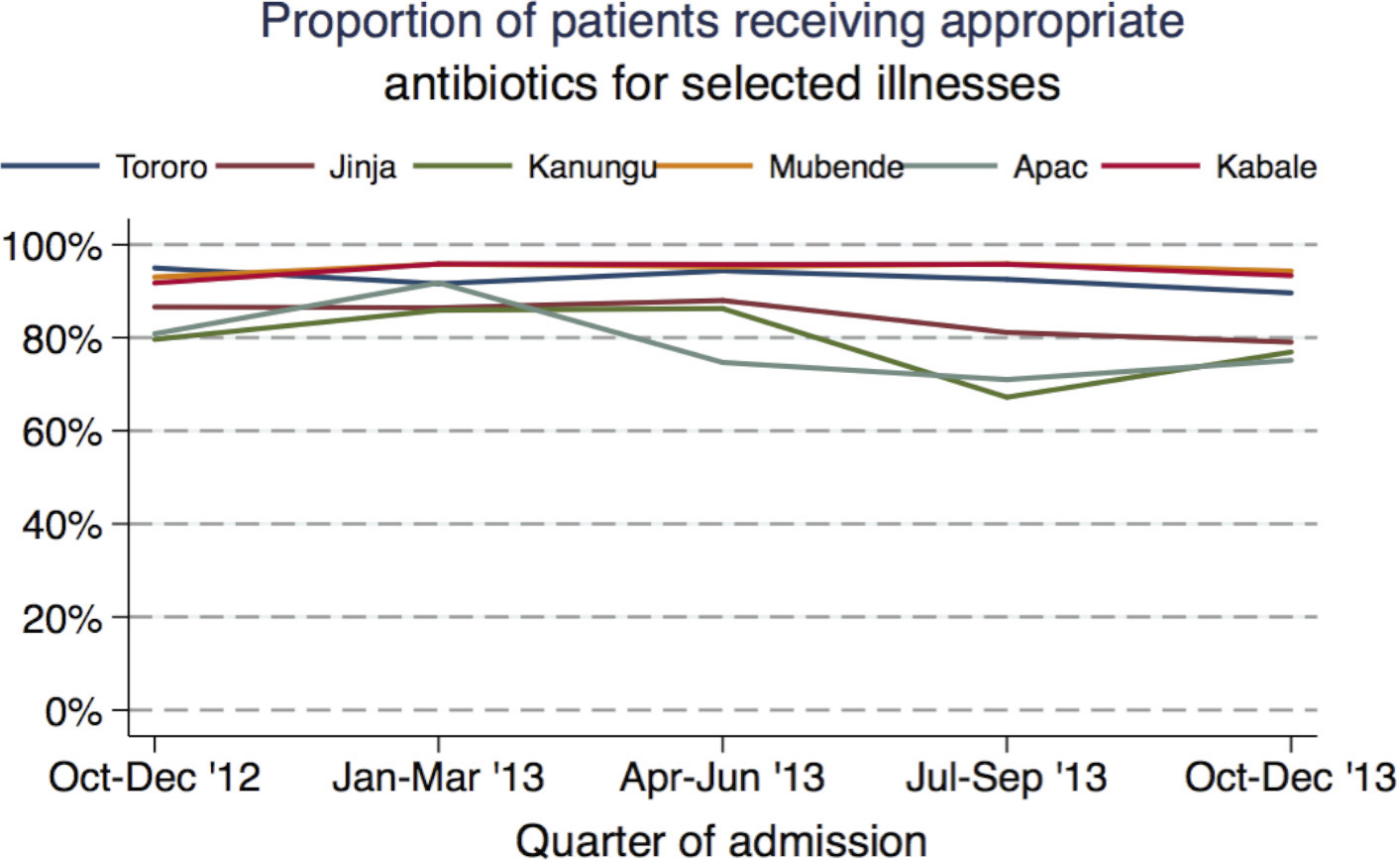
Trends in appropriate inpatient case management of selected illnesses requiring antibiotics, by health facility.

Selected illnesses requiring antibiotics were reported in 32% of patients (range 20%-63% across the health facilities): pneumonia was the most common (16%), followed by malnutrition (11%), sepsis (8%), meningitis (<1%), and tetanus (<1%). The administration of appropriate antibiotics was common when indicated for pneumonia (96%) and sepsis (96%), but less common for meningitis (83%), malnutrition (77%) and tetanus (53%) (Table 3). Appropriate antibiotics were given to 89% of all patients diagnosed with any number of the selected illnesses. The rates of appropriate antibiotic use at the health facilities ranged from 78% in Apac to 95% in Kabale (Table 5). Children < 1 year of age were more likely to receive appropriate management for selected illnesses requiring antibiotics than children ≥ 5 years (94% vs. 85%; p<0.01). Children who were hospitalized for < 1 day were less likely to receive appropriate antibiotics than those hospitalized for one day or longer (67% vs. 90%; p<0.01). A positive malaria diagnostic test was associated with a lower rate of appropriate antibiotic prescription compared to children who did not have a positive test (76% vs. 94%; p<0.01). Declines in the rate of appropriate antibiotic prescription at three-month intervals were noted in four of six health facilities: Jinja (OR 0.85; 95% CI 0.79–0.91; p<0.01), Kanungu (0.80; 95% CI 0.66–0.98; p = 0.03), Apac (OR 0.86; 95% CI 0.77–0.95; p = 0.01), and Tororo (OR 0.86; 95% CI 0.75–0.98; p = 0.02) (Fig 3). There was no significant change over time in Mubende or Kabale.

**Table 5:** Associations between characteristics and appropriate inpatient antibiotic prescription for selected illnesses^a^.

Category	Co-variable	Appropriate management (%)	Univariable		Multivariable	
			OR (95% CI)	p-value	OR (95% CI)	p-value
	Apac	682/869 (78%)	reference	—	reference	—
	Kanungu	268/335 (80%)	1.10 (0.80–1.50)	0.56	1.51 (1.06–2.15)	0.02
Health facility	Jinja	2,559/3,027 (85%)	1.50 (1.24–1.81)	<0.01	1.73 (1.39–2.14)	<0.01
	Tororo	1,724/1,857 (93%)	3.55 (2.80–4.52)	<0.01	4.46 (3.43–5.79)	<0.01
	Mubende	2,189/2,308 (95%)	5.04 (3.95–6.45)	<0.01	5.64 (4.32–7.36)	<0.01
	Kabale	1,128/1,188 (95%)	5.15 (3.80–7.00)	<0.01	3.43 (2.46–4.77)	<0.01
Gender	Male	4,705/5,249 (90%)	reference	—	reference	—
	Female	3,845/4,335 (89%)	0.91 (0.80–1.03)	0.14	0.97 (0.84–1.12)	0.66
Age	≥ 5 years	939/1,103 (85%)	reference	—	reference	—
	1 - < 5 years	4,401/5,079 (87%)	1.13 (0.94–1.36)	0.18	1.41 (1.15–1.75)	<0.01
	< 1 year	3,210/3,402 (94%)	2.92 (2.34–3.64)	<0.01	2.76 (2.16–3.53)	<0.01
Duration of hospitalization	> 3 days	4,359/4,580 (95%)	reference	—	reference	—
	3 days	1,671/1,869 (89%)	0.43 (0.35–0.52)	<0.01	0.48 (0.39–0.60)	<0.01
	1–2 days	2,227/2,695 (83%)	0.24 (0.20–0.29)	<0.01	0.28 (0.24–0.34)	<0.01
	< 1 day	293/440 (67%)	0.10 (0.08–0.13)	<0.01	0.07 (0.06–0.09)	<0.01
Day of admission	Weekday	6,809/7,657 (89%)	reference	—	reference	—
	Weekend	1,741/1,927 (90%)	1.17 (0.99–1.38)	0.07	1.13 (0.94–1.36)	0.18
Malaria diagnostic test result	Negative or missing	6,656/7,089 (94%)	reference	—	reference	—
	Positive	1,894/2,495 (76%)	0.21 (0.18–0.23)	<0.01	0.20 (0.17–0.23)	<0.01

^a^ Pneumonia, severe malnutrition, sepsis, meningitis, and tetanus

**Fig 4:**
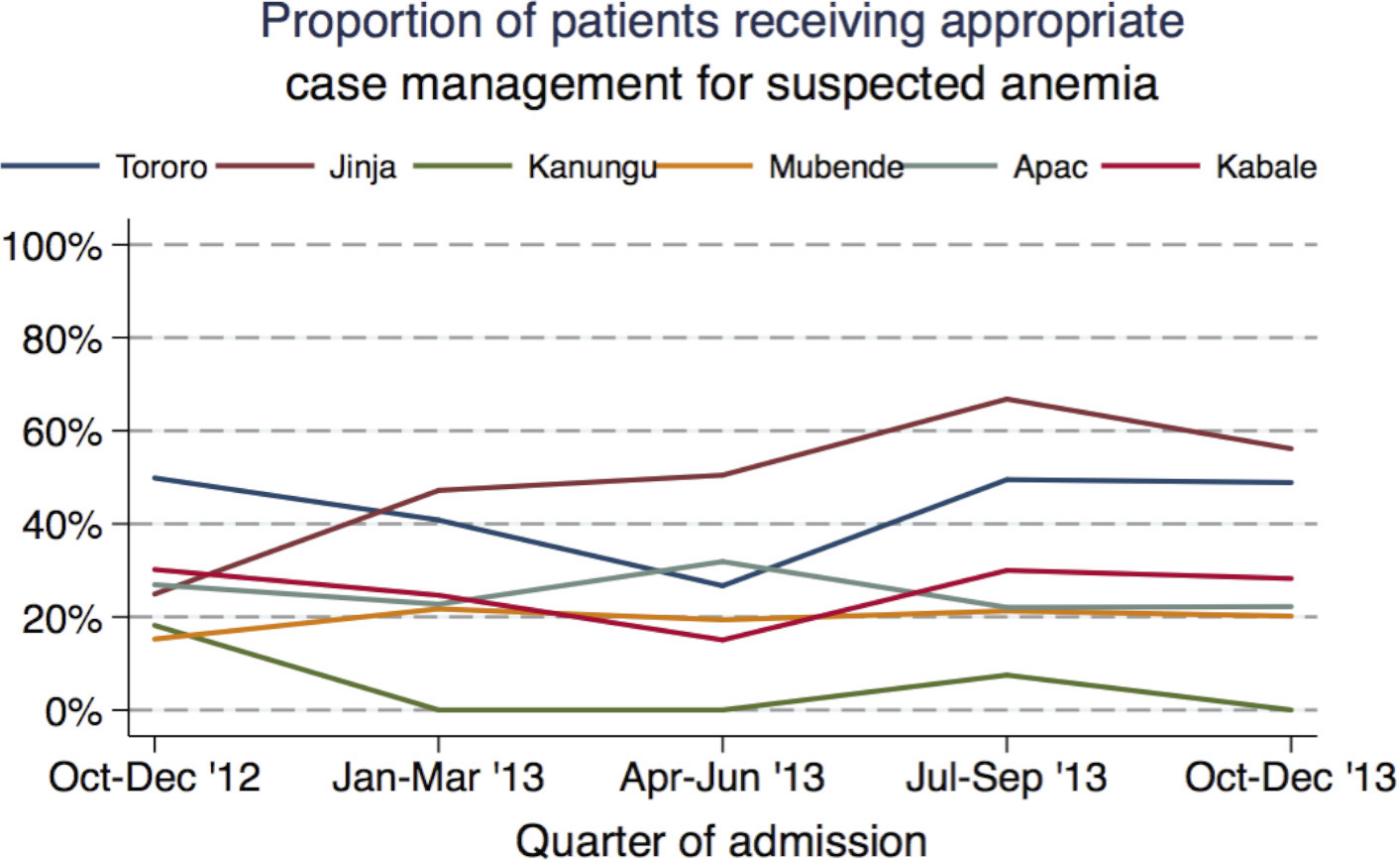
Trends in appropriate inpatient case management of suspected anemia, by health facility.

Anemia was suspected in 30% of patients (range 6%-46% across the health facilities), nearly all due to pallor (29%), compared to jaundice (2%), and sickle cell disease (2%). Hemoglobin testing was infrequently performed in patients with pallor (47%), jaundice (47%), and sickle cell disease (53%) (Table 3). Among the 4,183 anemia suspects who received hemoglobin testing, 2,355 (56%) met the criterion for severe anemia with a hemoglobin level < 5g/dl. Blood transfusions were administered to 66% of patients with severe anemia. Suspected anemia was correctly diagnosed and managed in 38% of applicable patients. The health facilities had different rates of appropriate case management for suspected anemia, from a low of 6% in Kanungu to a high of 49% in Jinja (Table 6). Children < 1 year of age were less likely to receive appropriate management for suspected anemia than children ≥ 5 years (35% vs. 40%; p<0.01). A positive malaria diagnostic test was associated with a higher rate of appropriate case management for suspected anemia (41% vs. 35%; p<0.01). A significant change in the rate of appropriate case management of suspected anemia was only observed in Jinja where management improved at three-month intervals (OR 1.42; 95% CI 1.36–1.49; p<0.01) (Fig 4).

**Table 6:** Associations between characteristics and receipt of appropriate inpatient case management for suspected anemia.

Category	Co-variable	Appropriate management (%)	Univariable		Multivariable	
			OR (95% CI)	p-value	OR (95% CI)	p-value
	Kanungu	5/95 (6%)	reference	—	reference	—
	Mubende	460/2,375 (19%)	4.32 (1.75–10.70)	<0.01	3.93 (1.59–9.74)	<0.01
Health facility	Kabale	101/411 (25%)	5.86 (2.32–14.83)	<0.01	6.31 (2.49–16.00)	<0.01
	Apac	70/279 (25%)	6.03 (2.35–15.44)	<0.01	5.39 (2.10–13.83)	<0.01
	Tororo	782/1,803 (43%)	13.79 (5.58–34.09)	<0.01	13.14 (5.31–32.52)	<0.01
	Jinja	2,043/4,199 (49%)	17.06 (6.92–42.06)	<0.01	16.89 (6.84–41.69)	<0.01
Gender	Male	1,897/4,966 (38%)	reference	—	reference	—
	Female	1,564/4,196 (37%)	0.96 (0.88–1.05)	0.36	0.96 (0.88–1.05)	0.37
Age	≥ 5 years	600/1,514 (40%)	reference	—	reference	—
	1 - < 5 years	2,073/5,387 (38%)	0.95 (0.85–1.07)	0.42	0.91 (0.80–1.03)	0.12
	< 1 year	788/2,261 (35%)	0.81 (0.71–0.93)	<0.01	0.75 (0.65–0.86)	<0.01
Duration of hospitalization	> 2 days	2,145/5,676 (38%)	reference	—	reference	—
	1–2 days	1,127/3,021 (37%)	0.98 (0.89–1.07)	0.66	0.78 (0.71–0.86)	<0.01
	< 1 day	189/465 (41%)	1.13 (0.93–1.37)	0.22	0.99 (0.81–1.21)	0.90
Day of admission	Weekday	2,743/7,157 (38%)	reference	—	reference	—
	Weekend	718/2,005 (36%)	0.90 (0.81–1.00)	0.04	0.83 (0.74–0.92)	<0.01
Malaria diagnostic test result	Negative or missing	1,621/4,636 (35%)	reference	—	reference	—
	Positive	1,840/4,526 (41%)	1.27 (1.17–1.39)	<0.01	1.29 (1.18–1.41)	<0.01

**Fig 5:**
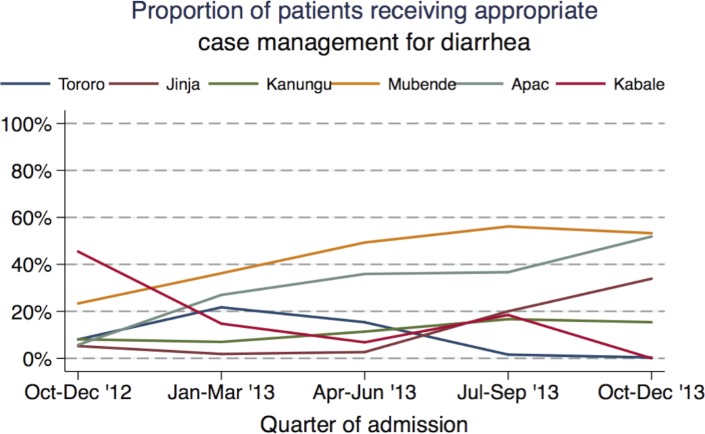
Trends in appropriate inpatient case management of diarrhea, by health facility.

Diarrhea was diagnosed in 12% of admissions (range 5–20% across the health facilities), with dysentery being diagnosed in a small proportion of all patients (<1%). Oral rehydration solution or intravenous fluids were administered to 63% of patients with diarrhea and antibiotics were administered 48% of patients with dysentery (Table 3). Zinc supplementation was infrequently administered (24%). All appropriate therapies were given to only 18% of patients with diarrhea, including dysentery. Appropriate case management of diarrhea ranged from 8% in Jinja to 44% in Mubende (Table 7). Children < 1 year of age and ages 1 to < 5 years were more likely to receive appropriate management for diarrhea than children ≥ 5 years (19% and 18% vs. 5%; p<0.01 for each comparison). A positive malaria diagnostic test was associated with a lower proportion of appropriate case management for diarrhea (11% vs. 20%; p<0.01). There was significant heterogeneity in changes in appropriate case management of diarrhea over time at the health facilities (Fig 5). Improvements were noted in Jinja (OR 2.48; 95% CI 1.91–3.21; p<0.01), Apac (OR 1.63; 95% CI 1.28–2.08; p<0.01), and Mubende (OR 1.34; 95% CI 1.21–1.48; p<0.01), while worsening was noted in Tororo (OR 0.61; 95% CI 0.54–0.71; p<0.01) and Kabale (OR 0.56; 95% CI 0.44–0.71; p<0.01).

**Table 7:** Associations between characteristics and receipt of appropriate inpatient case management for diarrhea.

Category	Co-variable	Appropriate management (%)	Univariable		Multivariable	
			OR (95% CI)	p-value	OR (95% CI)	p-value
	Jinja	52/664 (8%)	reference	—	reference	—
	Kanungu	18/192 (9%)	1.22 (0.69–2.14)	0.49	1.12 (0.64–1.97)	0.71
Health facility	Tororo	170/1,524 (11%)	1.48 (1.07–2.05)	0.02	1.49 (1.08–2.07)	0.02
	Kabale	54/354 (15%)	2.12 (1.41–3.18)	<0.01	1.90 (1.26–2.86)	<0.01
	Apac	58/194 (30%)	5.02 (3.30–7.62)	<0.01	4.89 (3.20–7.47)	<0.01
	Mubende	297/678 (44%)	9.17 (6.65–12.65)	<0.01	9.11 (6.58–12.62)	<0.01
Gender	Male	353/1,986 (18%)	reference	—	reference	—
	Female	296/1,620 (18%)	1.03 (0.87–1.22)	0.72	1.08 (0.90–1.30)	0.40
Age	≥ 5 years	8/170 (5%)	reference	—	reference	—
	1 - < 5 years	311/1,693 (18%)	4.56 (2.22–9.37)	<0.01	5.18 (2.47–10.84)	<0.01
	< 1 year	330/1,743 (19%)	4.73 (2.30–9.72)	<0.01	5.96 (2.84–12.47)	<0.01
Duration of hospitalization	> 5 days	116/517 (22%)	reference	—	reference	—
	1–5 days	520/3,011 (17%)	0.72 (0.57–0.90)	0.01	0.93 (0.72–1.19)	0.56
	< 1 day	13/78 (17%)	0.68 (0.37–1.30)	0.25	0.75 (0.38–1.48)	0.40
Day of admission	Weekday	528/2,832 (19%)	reference	—	reference	—
	Weekend	121/774 (16%)	0.81 (0.65–1.00)	0.05	0.87 (0.69–1.10)	0.23
Malaria diagnostic test result	Negative or missing	549/2,731 (20%)	reference	—	reference	—
	Positive	100/875 (11%)	0.51 (0.41–0.64)	<0.01	0.57 (0.44–0.72)	<0.01

## Discussion

Difficulty in assessing the quality of medical care in resource-limited health facilities has long hampered efforts to improve health care delivery [21–25]. Analyses often focus on inputs into the health facility—medications delivered, human resources, diagnostics available—and outputs in the form of clinical outcomes such as mortality and patient satisfaction. A more complete understanding of the quality of care in health facilities, however, requires a more nuanced view of how each patient and clinical situation is managed. Such a view requires robust patient-level data, such as that provided by the UMSP health facility-based inpatient surveillance program utilized in this study.

The principal findings from this study may help inform efforts to design interventions to improve inpatient health care quality in resource-limited settings. We found substantial heterogeneity in the rates of appropriate management across the four case management categories, from fairly high for suspected malaria (81%) and selected illnesses requiring antibiotics (89%), to very low for suspected anemia (38%) and diarrhea (18%). Within the composite indicators of quality, a few infrequently performed specific case management practices may be ideal targets for improving clinical outcomes. Improper diarrhea management was most influenced by low administration of zinc (24%), although oral or intravenous rehydration was also infrequently administered (63%), as were appropriate antibiotics for dysentery (48%). Zinc and rehydration solutions are of low-cost and widely available. Increasing the proportion of children with diarrhea who receive these interventions could affect mortality as has been shown in other settings [26–29]. Additionally, absent or incorrect antibiotic administration was influenced most by the failure to prescribe antibiotics to those with malnutrition (77%), a practice associated with a mortality reduction in a recent study [30]. Finally, improving the low rates of hemoglobin testing and blood transfusion for severe anemia (66%) could lead to lower inpatient mortality, especially if transfusions are performed quickly and in critically ill patients as described in an analysis from a Kenyan hospital [31].

Beyond the proportions of children receiving appropriate care for the selected case management categories, this study also reveals several key findings that merit further discussion: 1) differences in quality of care across the health facilities, 2) changes in quality care metrics over time, 3) the relatively high rates of appropriate malaria case management, and 4) the association between malaria diagnostic testing results and the management of non-malarial illnesses.

Individual health facilities were strongly associated with the odds of receiving appropriate case management for each composite indicator of quality care, but there were no consistent patterns of “high performing” or “low performing” health facilities when considering all four composite indicators together. Mubende Regional Hospital, for example, had the highest rate of appropriate management for diarrhea but was one of the lowest performing sites for the management of suspected anemia. Jinja Regional Hospital had the lowest rates of appropriate management for suspected malaria and diarrhea, but the highest rate of appropriate management for suspected anemia. Jinja’s inpatient staffing (pediatricians supervise interns who rotate through the pediatric wards) may play a role in these findings but further investigation is needed to determine how Jinja’s role as a teaching hospital affects the quality of care. The heterogeneity between sites also suggests that effective quality improvement initiatives will have to focus on deficiencies that are unique to each condition and health facility, rather than using a uniform approach across facilities.

Quality of care also varied over time at health facilities, but in different patterns. Among the four case management categories stratified across the six health facilities, six health facility-specific case management practices improved, six worsened, and 12 did not significantly change. Only two sites improved in two of the four case management categories (Jinja and Apac) yet both of these sites also saw declines in the rate of appropriate management of one category (selected illnesses requiring antibiotics). This finding suggests that the practice of quality care is dynamic at each health facility. Consequently, a better understanding of what is driving the fluctuation in quality care for specific case management practices will be important to ensure that improvements in care are consistent over time. The changes observed over time also highlight the limitation of cross-sectional surveys to measure case management practices and the effects of quality improvement interventions.

Given that UMSP interactions with the health facilities had focused on proper malaria diagnosis and treatment, data quality, and malaria diagnostic laboratory support at each facility for 1–2 years prior to the collection of data used in this study, we anticipated that malaria case management would show high rates of evidence-based best practices. Indeed we found that 97% of children with fever had malaria laboratory testing, compared to testing rates of 32% for children under the age of five presenting to Ugandan health facilities with fever in a large national survey [32] and 67% for suspected malaria cases seen in the public sector throughout Africa in 2012 [33]. Appropriate antimalarial prescription to children with a positive test was also high as 94% received appropriate therapy, similar to published data from other Ugandan hospitals [34]. Among the patients who did not receive appropriate case management, 71% of this failure was due to the prescription of antimalarials to those with a negative diagnostic test. This practice occurred in 29% of patients with fever and a negative test and was most common in Jinja, the site with the most clinicians and a high turnover of intern physicians who are responsible for care on the pediatric wards. Withholding antimalarials after a negative diagnostic test has proved to be a safe practice in a variety of settings [35–38] and has been recommended by WHO since 2010 [19]. This practice is particularly important as antimalarial prescription to patients with a negative diagnostic test or no diagnostic test can lead to unnecessary health expenditures and other diagnoses being ignored, which may be associated with increased mortality [39,40]. While the rate of inappropriate antimalarial prescription to those with a negative test in this study was suboptimal, the practice was observed less frequently than published reports in other inpatient and outpatient settings throughout Africa where rates of antimalarial prescription to those with a negative test prior to any training efforts have ranged from 48–70% for patients with fever [41–43], to as high as 95% for patients with a clinical diagnosis of malaria but a negative diagnostic test [34]. This relatively lower rate of inappropriate antimalarial prescription likely reflects the interventions associated with UMSP activities, an association that is supported by the 25% decrease in the proportion of febrile children with a negative test who were treated with antimalarials when data from the first two years of the study (April 2010 through March of 2012) are compared to data from the study period. Of note, the regional malaria transmission intensity did not seem to effect the quality of malaria care as the lowest performing site (Jinja) was in a region of medium transmission intensity and the highest performing sites (Tororo and Kabale) were in regions of high and low transmission intensity respectively.

Interestingly, a positive malaria diagnostic test had a large influence on the quality of management of other illnesses. Children with a positive malaria test were more likely to receive appropriate management of suspected anemia, but much less likely to receive appropriate management of selected illnesses requiring antibiotics and appropriate management of diarrhea. The strong association between malaria and anemia in children is well known and the finding of a positive malaria test may heighten the sensitivity of clinicians to the need to perform hemoglobin testing and blood transfusions. Conversely, a positive malaria test could distract clinicians from other co-morbid diagnoses that are not as strongly associated with malaria. The practice of incompletely managing other illnesses when a child has a positive malaria test is especially concerning as many individuals in areas of high malaria endemicity have asymptomatic parasitemia, including up to 30% of Ugandan schoolchildren involved in a study in Tororo District [44]. This finding suggests that a number of children admitted with a positive malaria diagnostic test, especially in highly endemic regions, were in fact hospitalized with illnesses other than malaria, which would necessitate different management. Additionally, severe malaria has been associated with a number of bacterial infections with rates of comorbid bacteremia and suspected pneumonia approximating 5% each from inpatient studies [45–48]. Quality improvement initiatives are thus needed to improve the delivery of treatments to patients with multiple diagnoses in Ugandan hospitals. To accomplish this, such initiatives will need to move beyond the common practice of focusing on a single disease and instead improve the rates of accurate diagnosis and management of patients with multiple clinical conditions.

This study has several limitations. With the exception of fever, malaria, severe anemia, and severe malnutrition, the diagnoses rendered were at the discretion of the clinician and could not be verified by any gold standard. Yet while some of these diagnoses may not be accurate, clinicians should still be conducting case management based on each patient’s presumptive diagnoses. Concerning the case management practices, many were broadly defined and there were a number of additional case management steps that were either not commonly done due to inadequate resources (such as lumbar puncture for meningitis) or not readily documented (such as nutritional support for malnutrition or tailored antibiotic selection for certain infections) (Table 1). Consequently these practices were not included as a measure of the quality of care, and this approach could have incorrectly classified some case management as appropriate at the level of the individual patient. This is especially true for non-malarial illnesses, which were not the initial focus of the MRF. Another limitation to our study was that diagnoses rendered and treatments given were derived entirely from a single data source, the MRF, and it is possible that correct or incorrect management was delivered to patients but not accurately documented on the MRF. UMSP, however, has conducted intensive efforts to promote high quality data collection with the aim of creating a more accurate and complete documentation of care relative to standard medical records at other government-run health facilities. Additionally, the MRF does not capture detailed information about the dosing of medications and an independent analysis would be needed to ensure that medications are dosed accurately. Notably, the health facilities involved in this study are not entirely representative of Ugandan hospitals due to their collaboration with UMSP. This collaboration likely influenced malaria care most significantly and provides a potential explanation for the relatively high rate of quality malaria case management. Finally, further studies will be needed to better understand the factors that are contributing to poor quality in certain case management practices. For one, it is not know how frequently key medications and medical supplies were not in stock, a factor which could significantly affect the practices of clinicians. Furthermore, provider knowledge, hospital staffing, and the different levels of training of clinicians could all be playing important roles in case management practices. Studies will also be needed to investigate the relationship between the quality of care and clinical outcomes such as mortality and disability.

With high quality data from 30,000 pediatric admissions, this study presents a detailed understanding of the quality of inpatient pediatric case management in Ugandan health facilities. The critical deficiencies identified in evidence-based best practices include the prescription of antimalarials to children with a negative malaria diagnostic test, low rates of antibiotic receipt for children with severe malnutrition, infrequent hemoglobin testing for suspected anemia, and sporadic use of rehydration solutions and zinc for diarrhea. Investigation of patient and health facility-level characteristics associated with these deficiencies reveals heterogeneity in quality between health facilities and the effect of age and malaria laboratory testing results on the management of different diagnoses. This highlights the importance of designing quality improvement initiatives that are customized to the individual deficiencies in care identified at each health facility and that address the management of multiple diseases at once. These findings represent an important step forward in efforts to improve pediatric care at health facilities in resource-limited settings. Given the immense burden of suffering and preventable deaths inflicted by the diseases described in this report, such efforts could have enormous potential to improve the health of children in Uganda and similar settings.

## Supporting Information

S1 Appendix Medical Record Form (MRF). (PDF)

## References

[1] Lozano R, Naghavi M, Foreman K, Lim S, Shibuya K, Aboyans V, et al Global and regional mortality from 235 causes of death for 20 age groups in 1990 and 2010: a systematic analysis for the Global Burden of Disease Study 2010. Lancet. 2012;380: 2095–128. 10.1016/S0140-6736(12)61728-0 23245604 PMC10790329

[2] UNICEF, World Health Organization, World Bank, United Nations. Levels and Trends in Child Mortality: Report 2013. New York, NY, USA; 2013.

[3] Nolan T, Angos P, Cunha AJ, Muhe L, Qazi S, Simoes EA, et al Quality of hospital care for seriously ill children in less-developed countries. Lancet. 2001;357: 106–10. 11197397 10.1016/S0140-6736(00)03542-X

[4] Jones G, Steketee RW, Black RE, Bhutta ZA, Morris SS, others. How many child deaths can we prevent this year? Lancet. 2003;362: 65–71. 12853204 10.1016/S0140-6736(03)13811-1

[5] English M, Esamai F, Wasunna A, Were F, Ogutu B, Wamae A, et al Assessment of inpatient paediatric care in first referral level hospitals in 13 districts in Kenya. Lancet. 2004;363: 1948–53. 15194254 10.1016/S0140-6736(04)16408-8

[6] English M, Esamai F, Wasunna A, Were F, Ogutu B, Wamae A, et al Delivery of paediatric care at the first-referral level in Kenya. Lancet. 2004;364: 1622–9. 15519635 10.1016/S0140-6736(04)17318-2

[7] Krug A, Pattinson RC, Power DJ. Why children die: an under-5 health care survey in Mafikeng region. South Afr Med J Suid-Afr Tydskr Vir Geneeskd. 2004;94: 202–6. 15098281

[8] Thomson N. Emergency medical services in Zimbabwe. Resuscitation. 2005;65: 15–9. 15797271 10.1016/j.resuscitation.2005.01.008

[9] Veirum JE, Biai S, Jakobsen M, Sandström A, Hedegaard K, Kofoed PE, et al Persisting high hospital and community childhood mortality in an urban setting in Guinea-Bissau. Acta Pædiatrica. 2007;96: 1526–30. 10.1111/j.1651-2227.2007.00467.x 17850399

[10] Reyburn H, Mwakasungula E, Chonya S, Mtei F, Bygbjerg I, Poulsen A, et al Clinical assessment and treatment in paediatric wards in the north-east of the United Republic of Tanzania. Bull World Health Organ. 2008;86: 132–9. 18297168 10.2471/BLT.07.041723 PMC2647389

[11] Gathara D, Opiyo N, Wagai J, Ntoburi S, Ayieko P, Opondo C, et al Quality of hospital care for sick newborns and severely malnourished children in Kenya: a two-year descriptive study in 8 hospitals. BMC Health Serv Res. 2011;11: 307 10.1186/1472-6963-11-307 22078071 PMC3236590

[12] Ralston ME, Day LT, Slusher TM, Musa NL, Doss HS. Global paediatric advanced life support: improving child survival in limited-resource settings. Lancet. 2013;381: 256–65. 10.1016/S0140-6736(12)61191-X 23332963

[13] Uganda Bureau of Statistics (UBOS), Macro International Inc., MEASURE Evaluation Uganda Child Verbal Autopsy Study 2007. Calverton, Maryland, USA: UBOS, Macro International Inc., and MEASURE Evaluation; 2008.

[14] Chandler CIR, Kizito J, Taaka L, Nabirye C, Kayendeke M, DiLiberto D, et al Aspirations for quality health care in Uganda: How do we get there? Hum Resour Health. 2013;11: 13 10.1186/1478-4491-11-13 23521859 PMC3610284

[15] Kiwanuka SN, Ekirapa EK, Peterson S, Okui O, Rahman MH, Peters D, et al Access to and utilisation of health services for the poor in Uganda: a systematic review of available evidence. Trans R Soc Trop Med Hyg. 2008;102: 1067–74. 10.1016/j.trstmh.2008.04.023 18565559

[16] Pariyo GW, Gouws E, Bryce J, Burnham G. Improving facility-based care for sick children in Uganda: training is not enough. Health Policy Plan. 2005;20: i58–68. 16306071 10.1093/heapol/czi051

[17] Uganda Ministry of Health. Uganda clinical guidelines 2012: national guidelines on management of common conditions. Kampala, Uganda; 2012.

[18] WHO. Pocket book of hospital care for children: guidelines for the management of common childhood illnesses- 2nd ed. Geneva, Switzerland: World Health Organization; 2013. 24006557

[19] WHO. Guidelines for the Treatment of Malaria (Second Edition). Geneva, Switzerland: World Health Organization; 2010 10.1186/1475-2875-9-212

[20] Centers for Disease Control and Prevention: Office of the Associate Director for Science. Distinguishing Public Health Research and Public Health Nonresearch. 2010. Available: http://www.cdc.gov/od/science/integrity/docs/cdc-policy-distinguishing-public-health-research-nonresearch.pdf

[21] Evans T, Stansfield S. Health information in the new millennium: a gathering storm? Bull World Health Organ. 2003;81: 856 14997237 PMC2572375

[22] AbouZahr C, Boerma T. Health information systems: the foundations of public health. Bull World Health Organ. 2005;83: 578–83. 16184276 PMC2626318

[23] Setel PW, Sankoh O, Rao C, Velkoff VA, Mathers C, Gonghuan Y, et al Sample registration of vital events with verbal autopsy: a renewed commitment to measuring and monitoring vital statistics. Bull World Health Organ. 2005;83: 611–7. 16184280 PMC2626308

[24] Gething PW, Noor AM, Gikandi PW, Ogara EA, Hay SI, Nixon MS, et al Improving imperfect data from health management information systems in Africa using space–time geostatistics. PLoS Med. 2006;3: e271 16719557 10.1371/journal.pmed.0030271 PMC1470663

[25] Nyamtema AS. Bridging the gaps in the Health Management Information System in the context of a changing health sector. BMC Med Inform Decis Mak. 2010;10: 36 10.1186/1472-6947-10-36 20579336 PMC2914713

[26] Victora CG, Bryce J, Fontaine O, Monasch R, others. Reducing deaths from diarrhoea through oral rehydration therapy. Bull World Health Organ. 2000;78: 1246–55. 11100619 PMC2560623

[27] Walker CLF, Black RE. Zinc for the treatment of diarrhoea: effect on diarrhoea morbidity, mortality and incidence of future episodes. Int J Epidemiol. 2010;39: i63–9. 10.1093/ije/dyq023 20348128 PMC2845862

[28] Srinivasan MG, Ndeezi G, Mboijana CK, Kiguli S, Bimenya GS, Nankabirwa V, et al Zinc adjunct therapy reduces case fatality in severe childhood pneumonia: a randomized double blind placebo-controlled trial. BMC Med. 2012;10: 14 10.1186/1741-7015-10-14 22316073 PMC3296597

[29] Munos MK, Walker CLF, Black RE. The effect of oral rehydration solution and recommended home fluids on diarrhoea mortality. Int J Epidemiol. 2010;39: i75–87. 10.1093/ije/dyq025 20348131 PMC2845864

[30] Trehan I, Goldbach HS, LaGrone LN, Meuli GJ, Wang RJ, Maleta KM, et al Antibiotics as Part of the Management of Severe Acute Malnutrition. N Engl J Med. 2013;368: 425–35. 10.1056/NEJMoa1202851 23363496 PMC3654668

[31] English M, Ahmed M, Ngando C, Berkley J, Ross A. Blood transfusion for severe anaemia in children in a Kenyan hospital. Lancet. 2002;359: 494–5. 11853798 10.1016/S0140-6736(02)07666-3

[32] Uganda Bureau of Statistics (UBOS), ICF International Inc. Uganda Demographic and Health Survey 2011. Kampala, Uganda and Calverton, Maryland: UBOS and ICF International Inc.; 2012.

[33] WHO. World Malaria Report 2013. Geneva, Switzerland: World Health Organization; 2013.

[34] Achan J, Tibenderana J, Kyabayinze D, Mawejje H, Mugizi R, Mpeka B, et al Case Management of Severe Malaria—A Forgotten Practice: Experiences from Health Facilities in Uganda. PLoS ONE. 2011;6: e17053 10.1371/journal.pone.0017053 21390301 PMC3046961

[35] Njama-Meya D, Clark TD, Nzarubara B, Staedke S, Kamya MR, Dorsey G. Treatment of malaria restricted to laboratory-confirmed cases: a prospective cohort study in Ugandan children. Malar J. 2007;6: 7 17239256 10.1186/1475-2875-6-7 PMC1797179

[36] D’ Acremont V, Malila A, Swai N, Tillya R, Kahama-Maro J, Lengeler C, et al Withholding Antimalarials in Febrile Children Who Have a Negative Result for a Rapid Diagnostic Test. Clin Infect Dis. 2010;51: 506–11. 10.1086/655688 20642354

[37] Mubi M, Janson A, Warsame M, Mårtensson A, Källander K, Petzold MG, et al Malaria Rapid Testing by Community Health Workers Is Effective and Safe for Targeting Malaria Treatment: Randomised Cross-Over Trial in Tanzania. Nosten F, editor. PLoS ONE. 2011;6: e19753 10.1371/journal.pone.0019753 21750697 PMC3130036

[38] Senn N, Rarau P, Manong D, Salib M, Siba P, Robinson LJ, et al Rapid diagnostic test-based management of malaria: an effectiveness study in Papua New Guinean infants with Plasmodium falciparum and Plasmodium vivax malaria. Clin Infect Dis. 2012;54: 644–51. 10.1093/cid/cir901 22198787

[39] Reyburn H. Overdiagnosis of malaria in patients with severe febrile illness in Tanzania: a prospective study. BMJ. 2004;329: 1212–0. 15542534 10.1136/bmj.38251.658229.55 PMC529364

[40] Opoka RO, Xia Z, Bangirana P, John CC. Inpatient Mortality in Children With Clinically Diagnosed Malaria As Compared With Microscopically Confirmed Malaria. Pediatr Infect Dis J. 2008;27: 319–24. 10.1097/INF.0b013e31815d74dd 18316995 PMC2607243

[41] Ssekabira U, Bukirwa H, Hopkins H, Namagembe A, Weaver MR, Sebuyira LM, et al Improved Malaria Case Management after Integrated Team-based Training of Health Care Workers in Uganda. Am J Trop Med Hyg. 2008;79: 826–33. 19052287

[42] Mangham LJ, Cundill B, Achonduh OA, Ambebila JN, Lele AK, Metoh TN, et al Malaria prevalence and treatment of febrile patients at health facilities and medicine retailers in Cameroon. Trop Med Int Health. 2011;17: 330–42. 10.1111/j.1365-3156.2011.02918.x 22098135

[43] Nyandigisi A, Memusi D, Mbithi A, Ang’wa N, Shieshia M, Muturi A, et al Malaria Case-Management following Change of Policy to Universal Parasitological Diagnosis and Targeted Artemisinin-Based Combination Therapy in Kenya. PLoS ONE. 2011;6: e24781 10.1371/journal.pone.0024781 21935464 PMC3173476

[44] Nankabirwa JI, Wandera B, Amuge P, Kiwanuka N, Dorsey G, Rosenthal PJ, et al Impact of Intermittent Preventive Treatment With Dihydroartemisinin-Piperaquine on Malaria in Ugandan Schoolchildren: A Randomized, Placebo-Controlled Trial. Clin Infect Dis. 2014;58: 1404–12. 10.1093/cid/ciu150 24621953 PMC4001293

[45] Bronzan RN, Taylor TE, Mwenechanya J, Tembo M, Kayira K, Bwanaisa L, et al Bacteremia in Malawian Children with Severe Malaria: Prevalence, Etiology, HIV Coinfection, and Outcome. J Infect Dis. 2007;195: 895–904. 17299721 10.1086/511437

[46] Bassat Q, Guinovart C, Sigaúque B, Mandomando I, Aide P, Sacarlal J, et al Severe malaria and concomitant bacteraemia in children admitted to a rural Mozambican hospital. Trop Med Int Health. 2009;14: 1011–9. 10.1111/j.1365-3156.2009.02326.x 19552643

[47] Berkley JA, Bejon P, Mwangi T, Gwer S, Maitland K, Williams TN, et al HIV Infection, Malnutrition, and Invasive Bacterial Infection among Children with Severe Malaria. Clin Infect Dis. 2009;49: 336–43. 10.1086/600299 19548833 PMC2853703

[48] Hendriksen ICE, Ferro J, Montoya P, Chhaganlal KD, Seni A, Gomes E, et al Diagnosis, Clinical Presentation, and In-Hospital Mortality of Severe Malaria in HIV-Coinfected Children and Adults in Mozambique. Clin Infect Dis. 2012;55: 1144–53. 22752514 10.1093/cid/cis590 PMC3447636

