# Supplementary material for: Quality of Inpatient Pediatric Case Management for Four Leading Causes of Child Mortality at Six Government-Run Ugandan Hospitals
Source: PLoS One. 2015 May 19;10(5):e0127192. doi: 10.1371/journal.pone.0127192 (PMC4437786; doi:10.1371/journal.pone.0127192)
Supplement: S1 Appendix — (PDF) [file pone.0127192.s001.pdf]

MOH-UMSP INPATIENT PAEDIATRIC SURVEILLANCE CASE RECORD FORM

**Date of admission:** |___|___| **day** |___|___| **month** |___|___| **year**		**IN PATIENT NUMBER:** ___________________________________	
**Time**	**of arrival in hospital:** |___|___| **hours** |___|___| **min** □ **am** □ **pm**	**Ward:**	**Bed number:**
	**of admission to ward:** |___|___| **hours** |___|___| **min** □ **am** □ **pm**		
**Patient's name: Last**______________________________**First**____			**Gender:** ☐ Male ☐ Female
**Age:** |___|___| **years** |___|___| **months** (If less than 5 years) |___|___| **days** (If less than 1 month)		**Re-admission (Admission of patient within 30 days of discharge from same hospital):** □ Yes □ No If yes, how many days: ________days	

ADDRESS AND NEXT OF KIN

**Village LCI:**	**Parish:**	**Sub-county:**
**District:**	**Name of LC1 chairman:**	
**Name of NOK:**	**Relationship with NOK:**	
**Name of father (if not NOK)**	**Telephone contact:**	
**Religion:**	**Tribe:**	

**Referring institution:**	□ Self referral	□ Health centre IV	□ Government hospital	□ Private hospital
	□ Private clinic	□ Other (specify)____		

HISTORY/SYMPTOM CHECK LIST

SYMPTOM (all must be answered)	PRESENT	SYMPTOM (all must be answered)	PRESENT	SYMPTOM (all must be answered)	PRESENT
**Fever**	□ Yes □ No	**Convulsions**	□ Yes □ No	**Diarrhoea**	□ Yes □ No
**Cough**	□ Yes □ No	**Altered consciousness**	□ Yes □ No	**Diarrhoea > 2 weeks**	□ Yes □ No
**Cough more than 2 weeks**	□ Yes □ No	**Vomiting**	□ Yes □ No	**Bloody diarrhoea**	□ Yes □ No
**Difficulty in breathing**	□ Yes □ No	**Unable to drink/breastfeed**	□ Yes □ No	**Passing tea coloured urine**	□ Yes □ No
**Other history:**		**Past medical history:**		**Feeding history:**	

**Immunization details**	□ None	□ Complete	□ Incomplete	□ On schedule	□ Not known

VITAL SIGNS

Temp: _____ °C	Weight: ________kg	Pulse: _____ / min	BP:_____/____ mmHg	Respirations: _____/min	Oxygen saturation: _____%

GENERAL EXAMINATION

**Pallor:** □ None □ Mild □ Mod □ Severe	**Severe wasting:** □ Yes □ No	**Sunken eyes** □ Yes □ No	**MUAC** _______cm
**Skin pinch return (sec):** □ 0 □ 1 □ 2	**Edema:** □ Yes □ No	**Jaundice** □ Yes □ No	**Height** _______cm

RESPIRATORY SYSTEM

**Deep breathing:** □ Yes □ No	**Airway:** □ Clear □ Strider
**Flaring of nostrils:** □ Yes □ No	**Wheezing:** □ Yes □ No
**Intercostal recession:** □ Yes □ No	**Pleural rub:** □ Yes □ No
**Subcostal recession:** □ Yes □ No	**Crackles:** □ Yes □ No

CARDIOVASCULAR SYSTEM

**Pulse:** □ Normal □ Weak	**Cap refill:** □ < 2s □ 2-3s □ > 3s

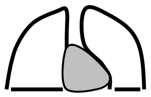

CENTRAL NERVOUS SYSTEM

**Unconscious:** □ Yes □ No	**Bulging fontanelle:** □ Yes □ No
**Lethargic:** □ Yes □ No	**Stiff neck:** □ Yes □ No
**Unable to sit/stand:** □ Yes □ No	**Kerning's sign:** □ Yes □ No

ABDOMINAL EXAMINATION

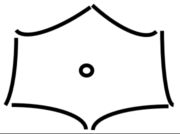

BCS: ________ **Eye Opening:** Spontaneous (1); Does not follow light (0) **Motor response**: Localizes pain (2); Unable to localize pain (1); No movement (0) **Verbal response:** Normal cry (2); Weak cry (1); No sound (0)

INITIAL LABORATORY RESULTS

**BS FOR MALARIA PARASITES** □ Positive □ Negative **TEST #**	**HIV TEST RESULTS** □ CTRR □ CTR **TEST #**	
**Malaria RDT:** □ Positive □ Negative	**Sickle Cell Test** □ Positive □ Negative	**Random Blood Sugar level:** ___________g/dl
**Haemoglobin testing desired:** □ Yes □ No<br>If Hb testing desired, was test done □ Yes □ No<br>If yes, result :_____________g/dl	**Others tests done:**	
If no, reasons: □ lab unable to do test (no reagents) □ patient declined	□ lab unable to do test (no equipment) □ Other, specify ________________________________	□ lab unable to do test (staff unavailable)
**Blood Group** □ A □ B □ AB □ O	□ **Rh Positive** □ **Rh Negative**	

ADMISSION DIAGNOSIS (Tick all that apply)

**Notifiable Diseases**	□ Leprosy	□ Tuberculosis – new case	□ Malignancy
□ Acute flaccid paralysis	□ Malaria – uncomplicated	**Diseases of the Newborn**	□ Sickle Cell Disease
□ Cholera	□ Malaria – severe/complicated	□ Birth asphyxia	**Malnutrition**
□ Dysentery	□ Meningitis (other)	□ Congenital malformation	□ Mild acute malnutrition
□ Guinea worm	□ Onchocerciasis	□ Hemorrhagic disease	□ Moderate acute malnutrition
□ Meningitis (meningococcal)	□ Peritonitis	□ Prematurity	□ Severe acute malnutrition + edema
□ Measles	□ Pyrexia of unknown origin	□ Respiratory distress syndrome	□ Severe acute malnutrition – edema
□ Tetanus (neonatal)	□ Schistosomiasis	**Non-Infectious Diseases**	**Medical Emergencies**
□ Plague	□ Septicaemia	□ Anaemia	□ Cerebro-vascular events
□ Rabies	□ Sleeping sickness	□ Animal/snake bite	□ Cardiac arrest
□ Yellow fever	□ Tetanus (over 28 days)	□ Asthma	□ Gastrointestinal bleeding
□ Viral haemorrhagic fever	□ Typhoid fever	□ Burns	□ Respiratory distress
**Other Infectious Diseases**	□ Urinary tract infection (UTI)	□ Childhood mental disorders	□ Renal failure – acute
□ AIDS	□ Otitis Media	□ Diabetes mellitus	□ Poisoning
□ Diarrhoea – acute	**Respiratory Diseases**	□ Epilepsy	**Other Diagnosis**
□ Diarrhoea – persistent	□ Pneumonia	□ Injuries – Road traffic accident	
□ Genital infections	□ Respiratory infections (other)	□ Injuries – Trauma, other cause	
□ Hepatitis	□ Tuberculosis – known case	□ Liver disease	

SUPPORTIVE CARE PLAN (Tick all that apply)

**BLOOD TRANSFUSION**			**OTHER FORMS OF SUPPORTIVE CARE**	
Is blood transfusion desired	□ Yes	□ No	□ IV Fluids: ________________ mL	
If yes, state reasons	□ Hb ≥ 5 but clinical indication	□ No Hb but clinical indication	□ Tepid sponging	□ Oxygen
	□ Hb < 5	□ Other, _________________	□ Left lateral position	□ Keep patient warm
If desired, was blood given: □ Yes □ No	If yes , volume_____ml		□ Nutritional rehabilitation	□ Nasogastric tube
Transfusion date: |__|__| **day** |__|__| **mo** |__|__| **yr**	Blood unit #:______________		□ Other (specify): _________________________	
If desired, but not given, state reasons	□ Blood not available	□ No blood grouping service	□ Other (specify): _________________________	
	□ Patient declined □ Patient died	□ Blood type not available	□ Other (specify): _________________________	

REASONS FOR DIAGNOSIS OF SEVERE/COMPLICATED MALARIA

□ Prostration	□ Haemoglobinuria	□ Hypoglycemia	□ Vomiting everything
□ Coma (deeply unconscious)	□ Convulsions (≥ 3 in 24 hours)	□ Acidosis	□ Convulsions (≤ 2 in 24 hours))
□ Respiratory distress	□ Circulatory collapse (shock)	□ Renal impairment	□ Inability to drink or breastfeed
□ Abnormal bleeding	□ Pulmonary oedema	□ Hyperlactataemia	□ Lethargic or unconscious
□ Jaundice	□ Severe anaemia	□ Hyperparasitemia	□ Other, specify

REASONS FOR ADMISSION WITH POSITIVE MALARIA TEST IF NOT FULFILLING CRITERIA FOR SEVERE/COMPLICATED

□ Co-morbidity	□ ACTs out of stock	□ Moderately sick child	□ Parental request
□ Can't access ACTs (Pharmacy closed)	□ Unable to return home due to time/distance	□ Other (specify)	

MEDICINES	PRESCRIPTION		DRUG ADMINISTRATION RECORD (completed by nurses or clinician)														
	Date	Dosing instructions	Date	Time	Initial	Date	Time	Initial	Date	Time	Initial	Date	Time	Initial	Date	Time	Initial
□ Quinine – IV																	
□ Artemether – IM																	
□ Artesunate – IV																	
□ Artesunate – rectal																	
□ Quinine – oral																	
□ SP – oral																	
□ Artesunate – oral																	
□ Chloroquine – oral																	
□ Coartem – oral																	
□ Amodiaquine – oral																	
□ Chloramphenicol – IV																	
□ Gentamicin – IV																	
□ Penicillin – IV																	
□ Ceftriaxone – IV																	
□ Ampicillin IV																	
□ Cotrimoxazole – oral																	
□ Nalidixic acid – oral																	
□ Amoxil – oral																	
□ Albendazole – oral																	
□ Ketaconazole – oral																	
□ Mebendazole – oral																	
□ TB drugs																	
□ Diazepam – rectal																	
□ ORS																	
□ Vitamin A																	
□ Zinc																	
□ Ferrous sulphate																	
□ Folic acid																	
□ Hydrocortisone IV/IM																	
□ Paracetamol																	
□ Other																	
□ Other																	
□ Other																	
□ Other																	

Name of admitting clinician: ____

DIAGNOSTIC TESTING OBTAINED AFTER ADMISSION				
|___|___|/|___|___|/|___|___| *day month year*	**BS:** □ Positive □ Negative	**RDT:** □ Positive □ Negative	**Hb level:** _____g/dl	**Other:**
|___|___|/|___|___|/|___|___| *day month year*	**BS:** □ Positive □ Negative	**RDT:** □ Positive □ Negative	**Hb level:** _____g/dl	**Other:**
|___|___|/|___|___|/|___|___| *day month year*	**BS:** □ Positive □ Negative	**RDT:** □ Positive □ Negative	**Hb level:** _____g/dl	**Other:**
|___|___|/|___|___|/|___|___| *day month year*	**BS:** □ Positive □ Negative	**RDT:** □ Positive □ Negative	**Hb level:** _____g/dl	**Other:**
|___|___|/|___|___|/|___|___| *day month year*	**BS:** □ Positive □ Negative	**RDT:** □ Positive □ Negative	**Hb level:** _____g/dl	**Other:**
|___|___|/|___|___|/|___|___| *day month year*	**BS:** □ Positive □ Negative	**RDT:** □ Positive □ Negative	**Hb level:** _____g/dl	**Other:**
|___|___|/|___|___|/|___|___| *day month year*	**BS:** □ Positive □ Negative	**RDT:** □ Positive □ Negative	**Hb level:** _____g/dl	**Other:**

FINAL DIAGNOSIS AT DISCHARGE (CHECK ALL THAT APPLY) – to be completed by the clinician at discharge			
**Notifiable Diseases**	□ Leprosy	□ Tuberculosis – new case	□ Malignancy
□ Acute flaccid paralysis	□ Malaria – uncomplicated	**Diseases of the Newborn**	□ Sickle Cell Disease
□ Cholera	□ Malaria – severe/complicated	□ Birth asphyxia	**Malnutrition**
□ Dysentery	□ Meningitis (other)	□ Congenital malformation	□ Mild acute malnutrition
□ Guinea worm	□ Onchocerciasis	□ Hemorrhagic disease	□ Moderate acute malnutrition
□ Meningitis (meningococcal)	□ Peritonitis	□ Prematurity	□ Severe acute malnutrition + edema
□ Measles	□ Pyrexia of unknown origin	□ Respiratory distress syndrome	□ Severe acute malnutrition – edema
□ Tetanus (neonatal)	□ Schistosomiasis	**Non-Infectious Diseases**	**Medical Emergencies**
□ Plague	□ Septicaemia	□ Anaemia	□ Cerebro-vascular events
□ Rabies	□ Sleeping sickness	□ Animal/snake bite	□ Cardiac arrest
□ Yellow fever	□ Tetanus (over 28 days)	□ Asthma	□ Gastrointestinal bleeding
□ Viral haemorrhagic fever	□ Typhoid fever	□ Burns	□ Respiratory distress
**Other Infectious Diseases**	□ Urinary tract infection (UTI)	□ Childhood mental disorders	□ Renal failure – acute
□ AIDS	□ Otitis Media	□ Diabetes mellitus	□ Poisoning
□ Diarrhoea – acute	**Respiratory Diseases**	□ Epilepsy	**Other Diagnosis**
□ Diarrhoea – persistent	□ Pneumonia	□ Injuries – Road traffic accident	
□ Genital infections	□ Respiratory infections (other)	□ Injuries – Trauma, other cause	
□ Hepatitis	□ Tuberculosis – known case	□ Liver disease	

FINAL OUTCOME AT DISCHARGE

**Date of discharge/death/referral/absconded** |___|___| **day** |___|___| **month** |___|___| **year**	Name of person completing discharge form:
**Time of discharge/death/referral/absconded** |___|___| **hours** |___|___| **min** □ **am** □ **pm**	

Disposition: (tick one)

□ Death – if yes, list cause(s) of death : ____

□ Improved with disability – if disability, list:____

□ Improved no disability

□ Absconded (Run away from hospital prior to official discharge)

□ Referred to____
